# Antioxidant Status in the Soleus Muscle of Sprague-Dawley Rats in Relation to Duodenal-Jejunal Omega Switch and Different Dietary Patterns

**DOI:** 10.1155/2018/3795070

**Published:** 2018-07-08

**Authors:** Bronisława Skrzep-Poloczek, Dominika Stygar, Elżbieta Chełmecka, Katarzyna Nabrdalik, Ewa Romuk, Jakub Poloczek, Tomasz Sawczyn, Konrad W. Karcz, Janusz Gumprecht

**Affiliations:** ^1^Department of Physiology, School of Medicine with the Division of Dentristy in Zabrze, Medical University of Silesia, Katowice, Poland; ^2^Department of Statistics, Department of Instrumental Analysis, School of Pharmacy with the Division of Laboratory Medicine in Sosnowiec, Medical University of Silesia, Katowice, Poland; ^3^Department of Internal Medicine, Diabetology and Nephrology in Zabrze, Medical University of Silesia, Katowice, Poland; ^4^Department of Biochemistry, School of Medicine with the Division of Dentistry in Zabrze, Medical University of Silesia, Katowice, Poland; ^5^Department of Rehabilitation, 3rd Specialist Hospital in Rybnik, Rybnik, Poland; ^6^Clinic of General, Visceral, Transplantation and Vascular Surgery, Hospital of the Ludwig Maximilian University, Munich, Germany

## Abstract

**Background:**

Obesity and chronic ingestion of lipid-rich meals are related to an enhanced oxidative stress (OS).

**Aims:**

To examine the influence of duodenal-jejunal omega switch surgery in combination with different diets on the antioxidative status in the soleus muscle of rats.

**Methods:**

After 8 weeks on a high-fat diet (HF) or control diet (CD), rats underwent duodenal-jejunal omega switch (DJOS) or SHAM (control) surgery. After surgery, for the next 8 weeks, half of DJOS/SHAM animals were kept on the same diet as before, and half had a changed diet. The total superoxide dismutase (SOD), catalase (CAT), glutathione peroxidase (GPx), glutathione-S-transferase (GST), and glutathione reductase (GR) activity as well as malondialdehyde (MDA) concentration were measured in the soleus of rats.

**Results:**

CAT and GPx activity were significantly lower after DJOS surgery versus SHAM, regardless of the type of diet. The activity of CAT, SOD, GR, CuZnSOD, and GPx was altered in the CD/HF or HF/CD groups. After DJOS, the lowest muscle concentration of MDA was observed in the CD/CD group and the highest in CD/HF.

**Conclusions:**

DJOS surgery significantly decreases the antioxidative system in soleus muscles of rats. CD/HF and HF/CD dietary patterns lead to an increase in antioxidative activity, while remaining on unchanged diet (CD or HF) is associated with a reduced oxidative stress.

## 1. Introduction

Obesity and comorbidities related to it pose a major challenge to public health systems and affected individuals worldwide. Even though there are many treatment methods available, the one that leads to sustained and clinically relevant body weight loss is bariatric surgery [1, 2]. What is even more important is that it is also currently the only obesity treatment modality with a proven mortality benefit [1]. Surgical treatment of obesity became a metabolic surgery because it not only leads to a reduction in body weight but also influences different metabolic pathways, with incretin hormones and diabetes type 2 resolution being two of the most studied areas up to date [3]. However, one must remember that obesity as well as chronic ingestion of lipid-rich meals are related also to an enhanced oxidative stress which in turn causes many unfavourable health consequences [4–7]. Oxidative stress (OS) is just a general term for the cellular damage being caused by an imbalance between prooxidants such as reactive oxygen species (ROS) and antioxidants in favour of the first one mentioned [8]. ROS are necessary for many physiological functions; however, to maintain a physiologically beneficial level of ROS antioxidants, which are enzymatic and nonenzymatic molecules, is crucial [9]. Markers indicative of oxidative stress include elevated measures of reactive oxygen species (ROS) and diminished antioxidant defence, associated with lower antioxidant enzyme levels [4, 5]. OS is also related to direct damage to lipids, with the production of end products of lipid peroxidation with malondialdehyde (MDA) being the most mutagenic one [10]. One of many organs that can be affected by OS associated with obesogenic conditions like obesity itself and high-fat diet (HFD) is the skeletal muscle. The high abundance of fat exceeds the ability of the muscle to oxidize this substrate, which leads to intramyocellular deposition and intermission of normal muscle function, with lipotoxicity as a consequence [11]. Scientists are trying to understand the influence of various aspects related to different treatment methods like diets or metabolic surgery procedures on humans using animal models. Regarding HFD diet, bariatric surgery, and OS, there have been only single studies related to models of oxidative stress markers performed; however, neither one took into consideration all three mentioned aspects in one experiment. In relation to HF diet, Auberval et al. proved the increase of tissue but not plasma oxidative stress among the studied animals [12] and Pinho et al. revealed that HFD induces skeletal muscle OS in rats [13]. Similarly, bariatric surgery procedures have been linked to a reduction in oxidative stress in rats [14]. DJOS is a relatively new technique; thus, an animal model, for exploring the long-term physiological effects and pathophysiological outcomes of this procedure, is still needed [15, 16]. DJOS is a type of bypass-like procedure, with proximal loop duodenoenterostomy, where the pylorus of the patients is saved. This type of surgery allows for direct hindgut stimulation [17, 18]. The biochemical mechanisms responsible for the pathophysiological traits of obesity, insulin resistance, and type 2 diabetes mellitus are incompletely understood. In the presented study, we aimed to assess the influence of duodenal-jejunal omega switch (DJOS) surgery in combination with different types of diet on antioxidant status in the soleus skeletal muscles of rats, which to our best knowledge have not been studied up to date, neither in humans nor in animals.

## 2. Materials and Methods

### 2.1. Animals and Diets

The study was performed in accordance with the Guide for the Care and Use of Laboratory Animals. Male Sprague-Dawley rats (Charles River Breeding Laboratories, Wilmington, Mass) aged 7 weeks, 200 ± 7 g, were housed in a 12 h light-dark cycle, 22°C, and 40–60% humidity. All rats had free access to water and rat food (Provimi Kliba AG, Kaiseraugst, Switzerland). The control group was maintained on ssniff® EF R/M. Obesity was induced by placing the animals on a high-fat diet (HF; 23.0 kJ/g, 59% fat, 27% carbohydrate, and 14% protein (EF RAT/E15744/ssniff Spezialdiäten GmbH) for an average of two months. Animals maintained on the HF diet were pair-fed (kcal) with the animals exposed to an ad libitum control diet. The energy content of the high-fat and standard diets were 5.04 and 3.59 kcal/g (20.1 and 15.0 kJ/g), respectively.

### 2.2. Experimental Design

**Figure 1:**
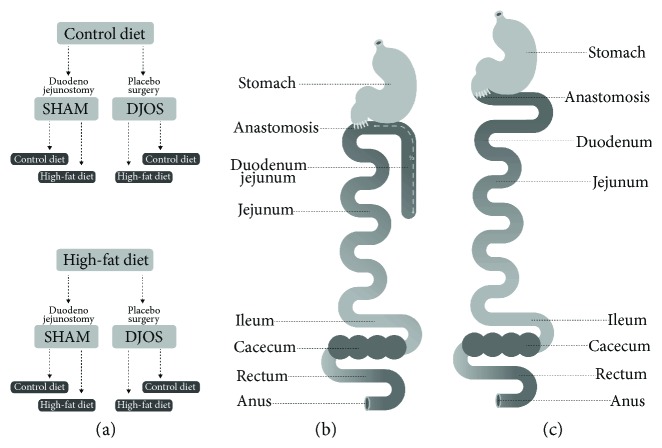
(a) Schematic illustration of DJOS and (b) SHAM surgery, respectively, and (c) scheme of experimental groups.

This individual study is based on an experimental design applied and described in an earlier work by Stygar et al. [19]. After one week of acclimatisation, the animals were assigned to the CD (*n* = 28) and HF groups (*n* = 28). After 8 weeks, both groups underwent the two different types of surgery SHAM, which is a control type of surgery (*n* = 14), and DJOS (*n* = 14), presented in Figure 1(a). After the surgery, the two groups of animals fed previously with CD or HF diets were divided further into 8 groups, depending on the postoperation diet regimen. In each surgery group, that is, SHAM and DJOS, 7 animals were kept on the same diet as before the surgery, and another 7 had the diet changed (Figure 1(a)). A number of rats were kept as small as possible in consideration of the “3Rs” for the humane treatment of animals [20]. All 7 rats survived in 7 out of 8 experimental groups. In HF/SHAM/CD, 6 out of 7 rats survived the experiment.

A DJOS and SHAM surgery was performed according to Karcz et al.'s methodology [16], described in the aforementioned study [19]. To perform DJOS surgery, the animals were anaesthetised with 2% isoflurane (AbbVie Deutschland GmbH & Co. KG, Ludwigshafen, Germany) and oxygen flow at 2 l/min under spontaneous breathing. Analgesia with xylazine (5 mg/kg, ip; Xylapan, Vetoquinol Biovet, Poland) and antibiotic prophylaxis with gentamicin were applied. The gastric volume was left intact, whereas the entire duodenum and the proximal jejunum were bypassed. The stomach was separated from the duodenum below the pylorus. The distal part of the transected duodenum was closed using Prolene 6/0 (Ethicon). The position of the duodenoenterostomy was determined to be at the aboral of the Treitz ligament, located approximately at one-third of the total small bowel length for DJOS. The duodenojejunostomy was performed as a simple antecolic, continuous end-to-side, hand-sewn, extramucosal anastomosis using 6-0 sutures. Postoperative analgesia was performed using carprofen (4 mg/kg, sc; Rimadyl, Pfizer, Switzerland) for 3 consecutive days after the surgery.

For the type of control operation called SHAM, transections and reanastomosis of the gastrointestinal tract were performed at the corresponding sites where enterotomies were performed for the duodenojejunostomy, thereby maintaining the physiological conduit of food passage through the bowel (Figures 1(b) and 1(c)).

### 2.3. Tissue Collection

After 16 weeks of the experiment and 8 weeks after the surgery, the tissue of the soleus muscle was harvested and the animals were euthanized. 100 mg of muscle tissue was homogenized in 1 ml of a homogenising buffer. All samples were snap frozen in liquid nitrogen and stored at −80°C until further analysis. All experimental procedures were approved by the Ethical Committee for Animal Experimentation of the Medical University of Silesia (58/2014). All applicable institutional and/or national guidelines for the care and use of animals were followed (Directive 2010/63/EU).

### 2.4. Oxidative Stress Marker Analysis

#### 2.4.1. Oxidative Enzyme Analysis

8 weeks after DJOS and SHAM surgery, an antioxidant system was analysed determining the activity of the following antioxidant enzymes in soleus muscle homogenates: glutathione reductase (glutathione-disulfide reductase, GR, and GSR), catalase (CAT), glutathione peroxidase (GPx), glutathione-S-transferase (GST), and total superoxide dismutase activity (SOD) and the nonenzymatic antioxidant system—lipid peroxidation by determining the malondialdehyde concentration.

#### 2.4.2. Glutathione Reductase Activity (EC 1.8.1.7)

GR enzymatic activity in the muscle homogenates was evaluated by a decrease in the concentration of NADPH in the samples using GR buffer (200 mM sodium phosphate pH 7.5, 6.3 mm EDTA), and kinetic reading was performed at a wavelength of 340 nm for 10 minutes [21].

#### 2.4.3. Catalase Activity (EC 1.11.1.6)

The catalase activity in muscle homogenates was measured using Aebi's methods. Briefly, 50 mM TRIS/HCl buffer, pH 7.4, and perhydrol were mixed with 50 *μ*l of homogenate. After 10 seconds, the absorbance was read at *λ* = 240 nm, every 30 seconds for 2 minutes. Enzymatic activity was expressed in IU/mg protein [22].

#### 2.4.4. Glutathione Peroxidase Activity (EC 1.11.1.9)

To measure the activity of glutathione peroxidase, the muscle homogenates were incubated with a GPx buffer (100 mM potassium phosphate with 1 mM EDTA pH 7.7), 40 mM sodium azide, GSH (diluted in 5% metaphosphoric acid), GR (GPx diluted in the buffer), NADPH (diluted with sodium bicarbonate 5%), and 0.5 mM tert-butyl. The decay of NADPH concentration was evaluated for 10 minutes in a spectrophotometer, at 340 nm [23].

#### 2.4.5. Glutathione-S-transferase Activity (EC 2.5.1.18)

Transferase activity of glutathione-S-transferase in muscle homogenates was estimated by the kinetic method, previously described by Habig and Jakoby [24]. 1-Chloro-2,3-dinitrobenzene was used as a substrate, and results are expressed in IU/g protein.

#### 2.4.6. Superoxide Dismutase Analysis (EC 1.15.1.1)

SOD isoenzyme activity was determined with the use of the spectrophotometric method by Oyanagui [25]. KCN was used as the inhibitor of the CuZnSOD isoenzyme. CuZnSOD activity was calculated as the difference between total SOD activity and MnSOD activity. SOD activity was calculated against a blank probe (containing bidistilled water). Enzyme activity was expressed as nitrite units (NU) per mg of protein in tissue. One NU exhibits 50% inhibition of formation of nitrite ion under the method's condition [25].

#### 2.4.7. Lipid Peroxidation

Malondialdehyde (MDA) concentration was measured in samples of muscle homogenates based on Ohkawa et al.'s method, using the reaction with thiobarbituric acid, with spectrophotometric detection employing 515 nm excitation and 552 nm emission wavelengths. MDA concentration was calculated from the standard curve, prepared from 1,1,3,3-tetraethoxypropane [26].

#### 2.4.8. Protein Concentration

Protein concentration was determined by Lowry methods using bovine serum albumin as the standard [27].

## 3. Statistical Analysis

Statistical analysis was performed using STATISTICA 12.5 PL (StatSoft, Cracow, Poland). Statistical significance was set at a *p* value below 0.05. All tests were two-tailed. Interval data were expressed as mean value ± standard deviation in the case of a normal distribution or as median/lower–upper quartile range in the case of data with skewed or nonnormal distribution. Distribution of variables was evaluated by the Shapiro-Wilk test and the quantile-quantile plot; homogeneity of variances was assessed by the Levene test. For comparison of data, the two-way parametric ANOVA with post hoc contrast analysis or nonparametric Kruskal-Wallis test or Mann–Whitney *U* test were used. In case of skewed data distribution, logarithmic transformation was done before analysis.

## 4. Results

The results of body weight change after DJOS and SHAM surgery in all experimental groups were previously presented by Stygar et al. [19]. The values of antioxidative systems for DJOS- and SHAM-operated groups are presented in Table 1.  Table 2 presents results of multiple comparisons in contrast analysis of DJOS- and SHAM-operated groups in relation to diet used before and after surgery. Column one presents a comparison between DJOS and SHAM surgery associated with different diets, column two shows comparisons between dietary groups of DJOS-operated animals, and column three shows comparisons between dietary groups of SHAM-operated animals.

### 4.1. Oxidative Enzyme Systems

#### 4.1.1. Glutathione Reductase

**Figure 2:**
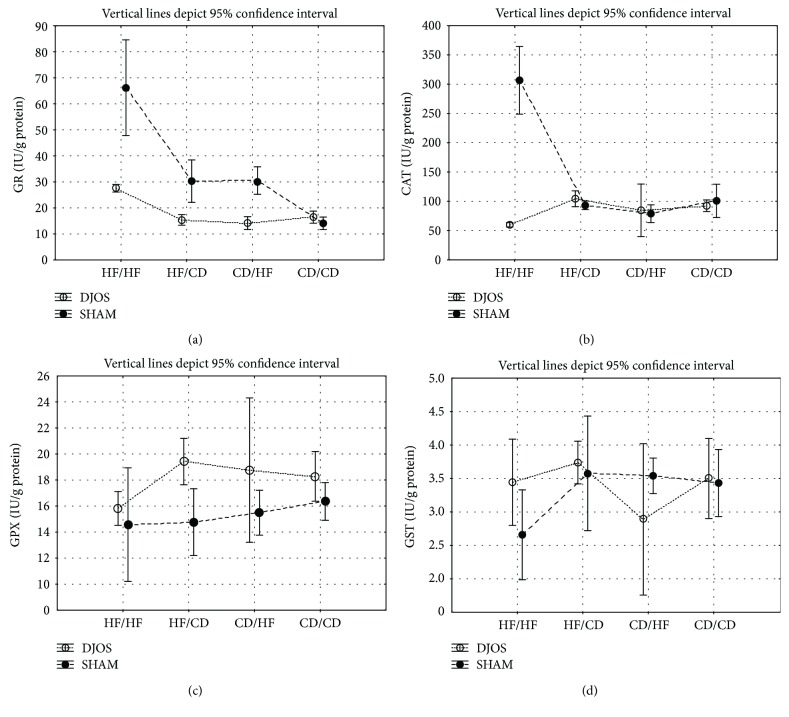
(a) Mean values of GR (IU/g) activity in four groups subjected to different dietary patterns, according to the DJOS and SHAM operation type. Statistical significance was set at *p* < 0.05. Vertical lines depict 95% confidence interval. DJOS: duodenal-jejunal omega switch surgery; HF: high-fat diet; CD: control diet; HF/HF, CD/HF, HF/CD, CD/CD: type of diet 8 weeks before/8 weeks after surgery. (b) Mean values of CAT (IU/g) activity in four groups subjected to different dietary patterns, according to the DJOS and SHAM operation type. Statistical significance was set at *p* < 0.05. Vertical lines depict 95% confidence interval. DJOS: duodenal-jejunal omega switch surgery; HF: high-fat diet; CD: control diet; HF/HF, CD/HF, HF/CD, CD/CD: type of diet 8 weeks before/8 weeks after surgery. (c) Mean values of GPX (IU/g) activity in four groups subjected to different dietary patterns, according to the DJOS and SHAM operation type. Statistical significance was set at *p* < 0.05. Vertical lines depict 95% confidence interval. DJOS: duodenal-jejunal omega switch surgery; HF: high-fat diet; CD: control diet; HF/HF, CD/HF, HF/CD, CD/CD: type of diet 8 weeks before/8 weeks after surgery. (d) Mean values of GST (IU/g) activity in four groups subjected to different dietary patterns, according to the DJOS and SHAM operation type. Statistical significance was set at *p* < 0.05. Vertical lines depict 95% confidence interval. DJOS: duodenal-jejunal omega switch surgery; HF: high-fat diet; CD: control diet; HF/HF, CD/HF, HF/CD, CD/CD: type of diet 8 weeks before/8 weeks after surgery.

GR muscle's activity in DJOS-operated animals compared to SHAM-operated ones was significantly different among HF/HF, HF/CD, and CD/CD diet groups (Figure 2(a), Tables 1 and 2). In the DJOS surgery group, GR muscle's activity in the HF/HF group was significantly lower when compared to the SHAM-operated HF/HF diet group. Oppositely, in the DJOS surgery group, GR muscle's activity in the HF/CD and CD/CD diet groups were significantly higher when compared to the SHAM-operated HF/CD and CD/CD diet groups of animals. There was no difference among the CD/HF diet group of DJOS- and SHAM-operated animals in relation to GR muscle's activity.

Taking into consideration DJOS surgery, there was a significant difference in terms of GR muscle's activity in the studied diet groups, namely, HF/HF and HF/CD, HF/HF and CD/HF, HF/HF and CD/CD, HF/CD and CD/HF, HF/CD and CD/CD, and CD/HF and CD/CD (Figure 2(a), Tables 1 and 2). The lowest GR muscle's activity was observed among the HF/HF diet group and the highest among the CD/CD group (Figure 2(a), Tables 1 and 2).

There were no significant differences in terms of GR muscle's activity in the studied diet groups in SHAM-operated animals.

#### 4.1.2. Catalase

CAT muscle's activity in DJOS-operated animals compared to SHAM-operated ones was significantly different among the HF/HF, HF/CD, CD/HF, and CD/CD diet groups (Figure 2(b), Tables 1 and 2). In the DJOS group, CAT activity was significantly lower in HF/HF, HF/CD, and CD/HF when compared to the HF/HF, HF/CD, and CD/HF diet groups of SHAM-operated animals. Oppositely, in the DJOS group, CAT activity was significantly higher in the CD/CD diet group when compared to the SHAM CD/CD diet group (Figure 2(b); Tables 1 and 2).

For the DJOS surgery, a significant difference in terms of CAT activity among the studied diet groups, namely, HF/HF and CD/HF, HF/CD and CD/HF, and CD/HF and CD/CD, was observed. The lowest CAT muscle activity was observed in the HF/HF diet group and the highest among the CD/HF diet group (Figure 2(b); Tables 1 and 2).

There was a significant difference in terms of CAT activity among the SHAM-operated studied diet groups, namely, HF/HF and CD/HF, HF/HF and CD/CD, HF/CD and CD/HF, and CD/HF and CD/CD (Figure 2(b); Tables 1 and 2), with the lowest value observed among the CD/CD group and the highest value among the CD/HF studied group (Figure 2(b); Tables 1 and 2).

#### 4.1.3. Glutathione Peroxidase Activity

GPx muscle's activity in DJOS-operated animals compared to SHAM-operated ones was significantly lowered among all analysed diet groups, namely, HF/HF, HF/CD, CD/HF, and CD/CD (Figure 2(c), Tables 1 and 2).

In DJOS-operated animals, there were significant differences in terms of GPx muscle's activity between each of the four diet groups, namely, HF/HF and HF/CD, HF/HF and CD/HF, HF/HF and CD/CD, HF/CD and CD/HF, HF/CD and CD/CD, and CD/HF and CD/CD, with the lowest value observed in the CD/CD group and the highest value in the CD/HF diet group (Figure 2(c), Tables 1 and 2).

In SHAM-operated animals, there were significant differences in terms of GPx muscle's activity between three out of four diet groups, namely, HF/HF and CD/HF, HF/HF and CD/CD, HF/CD and CD/CD, and CD/HF and CD/CD, with the lowest value observed among the CD/CD diet group and the highest one among the CD/HF diet group (Figure2(c); Tables 1 and 2).

#### 4.1.4. Glutathione-S-transferase Activity

GST muscle's activity in DJOS-operated animals compared to SHAM-operated ones was significantly lowered in HF/HF and CD/CD diet groups (Figure 2(d), Tables 1 and 2).

After DJOS surgery, there were significant differences in terms of GST muscle's activity between studied diet groups, namely, the HF/HF and CD/HF, HF/HF and CD/CD, HF/CD and CD/CD, and CD/HF and CD/CD diet study groups, with the lowest value observed among the CD/CD diet group and the highest ones among the CD/HF diet group (Figure 2(d), Tables 1 and 2).

There were no significant differences in terms of GST muscle's activity among any diet groups in SHAM-operated rats (Figure 2(d), Tables 1 and 2).

#### 4.1.5. Total Superoxide Dismutase Activity

**Figure 3:**
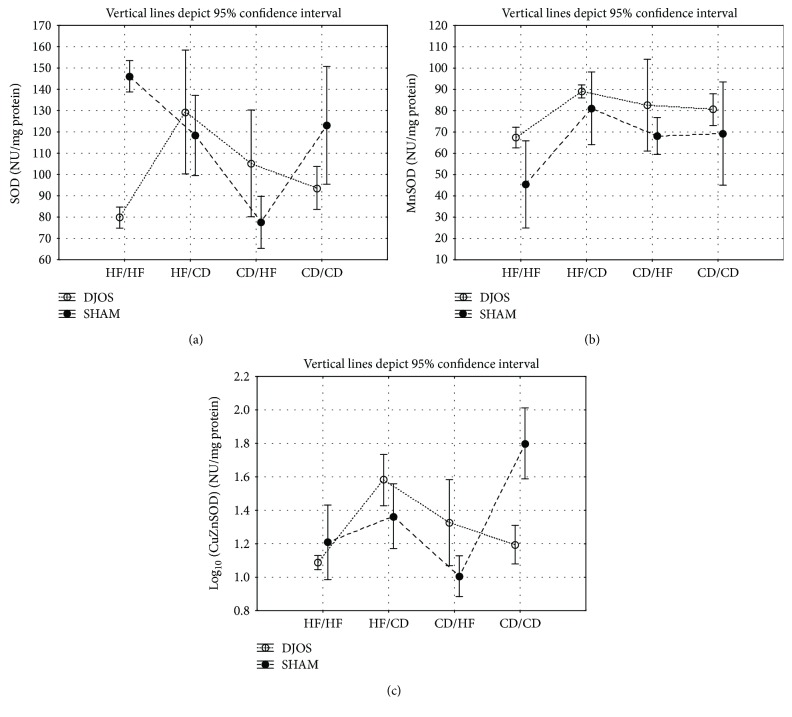
(a) Mean values of SOD (NU/mg) activity in four groups subjected to different dietary patterns, according to the DJOS and SHAM operation type. Statistical significance was set at *p* < 0.05. Vertical lines depict 95% confidence interval. DJOS: duodenal-jejunal omega switch surgery; HF: high-fat diet; CD: control diet; HF/HF, CD/HF, HF/CD, CD/CD: type of diet 8 weeks before/8 weeks after surgery. (b) Mean values of MnSOD (NU/mg) activity in four groups subjected to different dietary patterns, according to the DJOS and SHAM operation type. Statistical significance was set at *p* < 0.05. Vertical lines depict 95% confidence interval. DJOS: duodenal-jejunal omega switch surgery; HF: high-fat diet; CD: control diet; HF/HF, CD/HF, HF/CD, CD/CD: type of diet 8 weeks before/8 weeks after surgery. (c) Mean values of CuZnSOD (NU/mg) activity in four groups subjected to different dietary patterns, according to the DJOS and SHAM operation type. Statistical significance was set at *p* < 0.05. Vertical lines depict 95% confidence interval. DJOS: duodenal-jejunal omega switch surgery; HF: high-fat diet; CD: control diet; HF/HF, CD/HF, HF/CD, CD/CD: type of diet 8 weeks before/8 weeks after surgery.

SOD muscle's activity in DJOS-operated animals compared to SHAM-operated ones was significantly lowered in the HF/CD and CD/HF study diet groups (Figure 3(a), Tables 1 and 2).

After DJOS surgery, there were significant differences in terms of SOD muscle's activity between the studied diet groups, namely, HF/HF and CD/HF, HF/HF and CD/CD, HF/CD and CD/HF, and CD/HF and CD/CD, with the lowest value observed among the CD/CD diet group and the highest ones among the CD/HF diet group (Figure 3(a), Tables 1 and 2).

In SHAM-operated animals, there were significant differences in terms of SOD muscle's activity between four diet groups, namely, HF/HF and CD/CD, HF/CD and CD/CD, CD/HF, and CD/CD, with the lowest value observed among the CD/CD diet group (Figure 3(a), Tables 1 and 2).

#### 4.1.6. Mn Superoxide Dismutase Activity

MnSOD muscle's activity in DJOS-operated animals compared to SHAM-operated ones was significantly lowered in the CD/CD study diet group (Figure 3(b), Tables 1 and 2).

After DJOS, there were significant differences in terms of MnSOD muscle's activity between studied groups, namely, HF/HF and CD/HF, HF/HF and CD/HF, HF/CD and CD/HF, and CD/HF and CD/CD studied diet groups, with the lowest value observed among the HF/HF diet group (Figure 3(b), Table 2).

In SHAM-operated animals, there were significant differences in terms of SOD muscle's activity between the studied groups, namely, HF/HF and HF/CD, HF/HF and CD/HF, HF/HF and CD/CD, HF/CD and CD/HF, and CD/HF and CD/CD, with the lowest value observed among the HF/HF one (Figure 3(b), Tables 1 and 2).

#### 4.1.7. CuZn Superoxide Dismutase Activity

CuZnSOD muscle's activity in DJOS-operated animals compared to SHAM-operated ones was significantly different in all studied diet groups. Among HF/HF, CD/HF, and CD/CD diet groups of DJOS-operated animals, ZnCuSOD activity was significantly lower when compared to the HF/HF, CD/HF, and CD/CD diet groups of SHAM-operated animals (Figure 3(c), Tables 1 and 2). In the HF/CD diet group of DJOS-operated animals, ZnCuSOD activity was significantly higher when compared to the HF/CD diet group of SHAM-operated animals (Figure 3(c), Tables 1 and 2).

After DJOS surgery, there were significant differences in terms of CuZnSOD muscle's activity between the studied diet groups, namely, HF/HF and HF/CD, HF/HF and CD/HF, HF/HF and CD/CD, HF/CD and CD/CD, and CD/HF and CD/CD, with the lowest two values observed in the CD/CD diet group and the highest value in the HF/HF group (Figure 3(c), Tables 1 and 2).

After SHAM surgery, there were significant differences in terms of CuZnSOD muscle's activity between the studied diet groups, namely, HF/HF and HF/CD, HF/HF and CD/HF, HF/HF and CD/CD, HF/CD and CD/HF, and CD/HF and CD/CD, with the lowest value observed among the HF/CD diet group and the highest among the HF/HF studied group (Figure 3(c), Tables 1 and 2).

#### 4.1.8. Lipid Peroxidation

**Figure 4:**
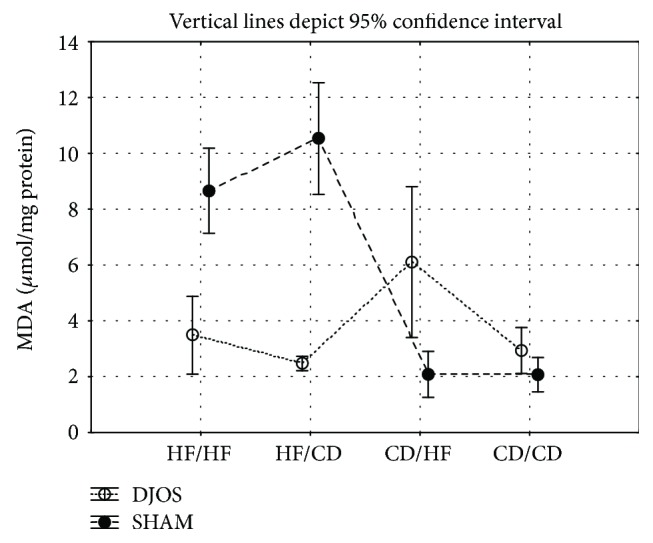
Mean values of MDA (*μ*mol/g) concentration in four groups subjected to different dietary patterns, according to the DJOS and SHAM operation type. Statistical significance was set at *p* < 0.05. Vertical lines depict 95% confidence interval. DJOS: duodenal-jejunal omega switch surgery; HF: high-fat diet; CD: control diet; HF/HF, CD/HF, HF/CD, CD/CD: type of diet 8 weeks before/8 weeks after surgery.

*(1) Malondialdehyde Concentration*. MDA muscle's activity in DJOS-operated animals compared to SHAM-operated ones was significantly lowered in the HF/CD and CD/CD study diet groups (Figure 4, Tables 1 and 2).

After DJOS surgery, there were significant differences in terms of MDA muscle's concentrations between the studied groups, namely, HF/HF and HF/CD, HF/HF and CD/HF, HF/HF and CD/CD, HF/CD and CD/CD, and CD/HF and CD/CD, with the lowest value observed in the CD/CD diet group and the highest in the CD/HF diet group (Figure 4, Tables 1 and 2).

After SHAM surgery, there were significant differences in terms of MDA muscle's activity between the studied groups, namely, HF/HF and HF/CD, HF/HF and CD/HF, HF/CD and CD/HF, HF/CD and CD/CD, and CD/HF and CD/CD, with the lowest value observed in the CD/CD diet group and the highest value in the HF/CD diet group (Figure 4, Tables 1 and 2).

## 5. Discussion

It is already known that both high-fat diet and/or obesity lead to an increased OS [28]. It is however not known whether different types of diet combined with bariatric surgery decrease the skeletal muscles' OS. In this study, we have, for the first time, proven that DJOS surgery positively reduced the redox state of the rat's soleus muscle cells in comparison to the SHAM procedure. It was also noticed that the type of diet applied before and after DJOS and SHAM surgery is associated with the different levels of OS status, antioxidant enzymatic activity, and nonenzymatic system in the soleus muscle of rats. Thus, the antioxidant activity was related to the type of diet before and after the surgery and the studied enzyme. Treatment with the control dietary pattern or HF diet, both before and after surgery, was associated with the lowest antioxidants' activity, while changing the diet from CD to HF or the reverse significantly increased the ROS generation and lipid peroxidation measured by MDA concentration. Because there were no studies performed up to date that took under consideration the association of bariatric surgery in relation to diet with skeletal muscle's antioxidant system activity, we will refer to studies examining single components of our experiment in this discussion section.

Metabolic disorders are associated with alterations in lipid and glucose metabolism in skeletal muscle. Some of those alterations are explained by defects in insulin signalling, glucose transport, or glycogen synthesis in skeletal muscles [29, 30]. This leads to deterioration of metabolic function and is associated with mitochondrial dysfunction [31–33]. It is known that altered mitochondrial function in skeletal muscle leads to reduced fatty acid oxidation and reduction of insulin-stimulated glucose transport. This may be understood as a hallmark of insulin resistance and diabetes mellitus type 2 [34]. Under obesogenic conditions, for example, high-fat diet, the ability of muscle tissue to oxidize the fat content is highly reduced and may lead to an increased level of ROS; thus, ROS production is intensified under inflammatory conditions characteristic for obesity. ROS, by its dual nature, may play a pathogenic role in many muscular diseases or lead to further injury by oxidatively damaging differentiating myoblasts and myotubes [31]. Conversely, together with other factors such as growth factors and chemokines, ROS takes part in a process of muscle regeneration and repair [31]. ROS also stimulates signalling pathways relevant to skeletal muscle metabolism, homeostasis, and adaptation [35]. The muscle cellular antioxidant capacity is the main factor, which can curb the negative activity of ROS, and the modulation of muscle sensitivity to ROS depends on the ROS level. The antioxidant enzymes keep ROS on the physiological level, where they can act as defence systems, signalling molecules, and mitochondrial function modulators [35, 36].

In our study, the CAT and GPx activity was significantly lower after DJOS surgery in comparison with the SHAM one in all diet study groups. It was not dependent on diet; nevertheless, the redox state of the muscle cells and activity of CAT, SOD, and GPx was altered in the groups maintained on the mixed CD/HF diet. GR and CuZnSOD activity was increased after DJOS surgery compared to the SHAM one among animals fed with the HF diet before the surgery and CD following the procedure. These enzymatic antioxidants are the main force of prevention mechanisms, controlling the formation of ROS [37, 38]. Apart from ATP production, mitochondrial areas are major sites for ROS production. Characteristic of rat soleus muscles are mitochondria-rich slow-twitch type I fibres, which exhibit oxidative metabolism and fatigue resistance [39]. Long-term exposure to an HF diet increases the muscle rates of fatty acid oxidation regardless of the fibre type [13]. Chronic disturbances in energy metabolism caused by excessive food intake and obesity lead to an increase in ROS production and mitochondrial dysfunction [34]. The changes in the structure and functions of mitochondria in the skeletal muscles of obese mice favour the generation of ROS, the development of oxidative stress, and insulin resistance [40, 41]. Chronic oxidative stress and inflammation mainly derived from reduced mitochondrial mass, changed morphology, and mitochondrial metabolic dysfunction are proven to induce lipid accumulation and insulin resistance in muscle tissue [34, 42–44]. Recent laboratory findings prove that a high-fat diet changes the mitochondrial structure in mice and the optimal mitochondrial function is obtained only under the condition of caloric restriction or regular diet [34]. Our findings show that DJOS surgery reduces the deleterious impact of an HF/HF diet on OS but does not act efficiently under the condition of a change in dietary pattern. Other studies confirm the reduction in oxidative stress and a downregulation of antioxidant enzymes after duodenojejunostomy in the insulin resistance and diabetic animal models [14].

In this study, the HF diet, applied before and after surgery, stimulated antioxidative activity less than the CD/HF and HF/CD ones. Here, rats were exposed to an HF diet for 16 weeks, and this could have allowed enough time for the antioxidant system to adapt to a proinflammatory diet and quench ROS production. In this manner, it may be interpreted as an increase in the oxidative capacity of the soleus muscle. After DJOS surgery the activity of Mn SOD, CAT, GPx, GR, and GST was reduced in the presence of the HF/HF diet when compared to the HF/CD and CD/HF dietary groups. Significant differences in the activity of all studied enzymes were observed between the HF/HF and CD/HF groups, with higher activity of CAT, GPx, GST, Mn SOD, and SOD and MDA concentration after the change of the diet from CD to HF. The activity of studied enzymes and MDA concentration was significantly higher also in the CD/HF dietary pattern when compared to CD/CD, with the exception of GR. The different activity profile of ZnCu SOD, GR, and total SOD, when compared to other studied enzymes and MDA concentration, could have been understood as a compensation mechanism, reducing the potentially deleterious impact of ROS production on soleus muscle metabolism and function under obesogenic conditions. It can also reflect on the intrinsic differences between the studied dietary patterns, in relation to the activity of antioxidative/prooxidative systems.

The change of the diet from CD to HF or HF to CD caused more disturbances in oxidative stress, measured by enzymatic activity and MDA concentration, than remaining on the same nutritional profile. We suggest that the change of a diet after 8 weeks, and hence the consumption of a different type of nutrients, influenced the biochemical pathways, causing perturbations and irregularities in substrate catabolism, which is also manifested in ROS production and antioxidative activity. Therefore, in relation to the general data presented above, it can be hypothesised that the way DJOS influences the soleus muscle's antioxidative enzyme activity is related to the type of diet implemented before and after the surgery and the type of enzyme studied. Changing the diet from CD to HF or vice versa seems to be the less preferable method of treatment, and remaining on the stable diet, preferably the control one, which is more favourable in terms of antioxidative enzyme activity following bariatric surgery would be the better option.

To our best knowledge, there has been no study performed to date that examined MDA skeletal muscles' activity following a bariatric surgery procedure in relation neither to HF or CD nor to animals or humans. Reduction of MDA skeletal muscle's activity following the bariatric surgery procedure stays in line with the recently performed study in humans by Monzo-Beltran et al. However, the MDA activity was measured in serum, not in skeletal muscle [45]. These authors observed that MDA progressively decreased in patients undergoing laparoscopic sleeve gastrectomy [45]. Even though bariatric surgery procedures have an advantage in decreasing lipid peroxidation measured with MDA skeletal muscle's concentration, the changes in the dietary pattern (CD/HF; HF/CD) are less favourable in comparison with HF/HF and CD/CD patterns. In the light of the presented studies, it might be reflected that bariatric procedure has an advantageous effect in terms of mobilisation of antioxidative systems and OS reduction. To our knowledge, the phenomenon of diverse responses to OS amplified by HF diet across different muscle types has been studied [13]. Therefore, the observations coming from soleus muscle are not necessarily reflective of the redox response to diet and bariatric procedures in all skeletal muscles. Thus, further studies are needed to more widely explore our initial findings.

## 6. Conclusions

The dietary patterns applied for this research included combinations of the same diet before and after surgery (HF/HF, CD/CD) as well as different ones (HF/CD, CD/HF). After 16 weeks of the experiment and 8 weeks after DJOS and SHAM surgery, we observed the following: (i) Enzymatic systems represented by GPX, CAT, CuZnSOD, and nonenzymatic MDA showed a significantly lower level of activity and concentration in muscle after DJOS surgery in comparison to SHAM-operated animals. (ii) GR showed a significantly increased activity after DJOS in relation to SHAM operation. This may suggest a strong beneficial impact of DJOS surgery on the dynamics of antioxidative/oxidative processes. (iii) A change in diet, regardless of the type of diet, stimulated OS in DJOS-operated rats. (iv) For most of the analysed parameters, we observed that the same type of diet before and after surgery, which was HF/HF and CD/CD, induced OS less than a change in dietary pattern from HF to CD or from CD to HF.

We conclude that metabolic surgery together with mixed dietary patterns may be potentially used as a strategy to modulate oxidative stress under pathological conditions. Long-term application of mixed control and obesogenic dietary patterns led to significant changes after DJOS surgery, many times reducing its beneficial effect, measured by selected antioxidants.

## Figures and Tables

**Table 1:** Antioxidant activity and concentration levels in soleus muscle 8 weeks after DJOS (1st column) and SHAM (2nd column) surgery, subjected to 16 weeks of different dietary patterns, and intergroup comparison between DJOS and SHAM study groups (3rd column) using descriptive statistics and results of two-way analysis of variance. Statistical significance was set at *p* < 0.05.

	DJOS				SHAM				*p* ANOVA		
Parameter	HF/HF	HF/CD	CD/HF	CD/CD	HF/HF	HF/CD	CD/HF	CD/CD	Group	Op.	Int.
GR (IU/g)	3.61 ± 0.80	14.32 ± 2.94	7.72 ± 1.18	29.90 ± 8.98	9.96 ± 0.81	9.95 ± 0.93	6.57 ± 1.28	8.05 ± 2.63	<0.001	<0.001	<0.001
CAT (IU/g)	31.95 ± 4.57	34.65 ± 3.15	47.31 ± 3.45	39.28 ± 6.63	56.38 ± 13.99	58.93 ± 3.68	63.91 ± 2.19	29.00 ± 3.33	<0.001	<0.001	<0.001
GPX (IU/g)	2.37 ± 0.64	3.27 ± 0.87	4.44 ± 1.27	1.34 ± 0.14	3.26 ± 0.78	5.14 ± 1.02	8.84 ± 0.42	4.43 ± 0.25	<0.001	<0.001	<0.001
GST (IU/g)	1.12 ± 0.08	1.42 ± 0.26	1.47 ± 0.34	0.78 ± 0.09	1.57 ± 0.34	1.38 ± 0.32	1.49 ± 0.34	1.30 ± 0.11	<0.01	<0.01	<0.05
Total SOD (NU/mg)	85.59 ± 2.90	80.72 ± 6.0	105.64 ± 17.55	74.33 ± 1.69	93.16 ± 7.01	93.87 ± 3.24	91.53 ± 4.40	75.55 ± 1.27	<0.001	0.359	<0.001
MnSOD (NU/mg)	24.50 ± 4.05	36.00 ± 3.02	37.67 ± 11.22	27.59 ± 3.36	30.25 ± 2.93	40.93 ± 8.56	38.72 ± 8.33	39.44 ± 5.17	<0.001	<0.01	0.246
CuZnSOD (NU/mg)	55.49 ± 3.42	48.73 ± 1.63	43.30 ± 2.04	35.00 ± 2.92	72.19 ± 8.47	39.97 ± 4.56	53.93 ± 5.64	42.44 ± 5.61	<0.001	<0.001	<0.001
MDA (*μ*mol/g)	4.56 ± 0.24	5.49 ± 1.34	5.57 ± 0.24	3.41 ± 0.30	4.76 ± 0.57	6.82 ± 0.67	5.92 ± 0.36	4.45 ± 0.30	<0.001	<0.001	0.083

GR: glutathione reductase; CAT: catalase; GPX: glutathione peroxidase; SOD: total superoxide dismutase; GST: glutathione-S-transferase; MnSOD: Mn superoxide dismutase; ZnSOD: Zn superoxide dismutase; MDA: malondialdehyde; DJOS: duodenal-jejunal omega switch surgery; HF: high-fat diet; CD: control diet; HF/HF, CD/HF, HF/CD, CD/CD: type of diet 8 weeks before/8 weeks after surgery; Op.: operation type; Int.: interaction between group and operation type. Mean ± standard deviation or median (lower – upper quartile).

**Table 2:** Multiple comparisons in contrast analysis. Column 1: Inter-group comparisons between HF/HF, CD/HF, HF/CD, CD/CD groups DJOS versus SHAM; Column 2: Intragroup comparisons between HF/HF, CD/HF, HF/CD, CD/CD groups after DJOS surgery; Column 3: Intragroup comparisons between HF/HF, CD/HF, HF/CD, CD/CD groups after SHAM surgery. Post hoc analysis, statistical significance was set at a *p* < 0.05.

Post hoc	DJOS versus SHAM				DJOS						SHAM					
	1: HF/HF	2: HF/CD	3: CD/HF	4: CD/CD	1 versus 2	1 versus 3	1 versus 4	2 versus 3	2 versus 4	3 versus 4	1 versus 2	1 versus 3	1 versus 4	2 versus 3	2 versus 4	3 versus 4
GR (IU/g)	<0.01	<0.05	0.573	<0.001	<0.001	<0.05	<0.001	<0.01	<0.001	<0.001	0.993	0.090	0.333	0.104	0.355	0.472
CAT (IU/g)	<0.001	<0.001	<0.001	<0.01	0.479	<0.001	0.059	<0.01	0.227	<0.05	0.488	<0.05	<0.001	0.195	<0.001	<0.001
GPX (IU/g)	<0.05	<0.001	<0.001	<0.01	<0.05	<0.001	<0.05	<0.05	<0.001	<0.001	<0.001	<0.001	<0.01	<0.001	0.118	<0.001
GST (IU/g)	<0.01	0.799	0.909	<0.01	0.068	<0.05	<0.05	0.721	<0.001	<0.001	0.145	0.559	0.070	0.469	0.622	0.227
Total SOD (NU/mg)	0.072	<0.01	<0.01	0.776	0.259	<0.001	<0.05	<0.001	0.141	<0.001	0.862	0.694	<0.001	0.585	<0.001	<0.001
MnSOD (NU/mg)	0.118	0.193	0.780	<0.01	<0.01	<0.01	0.413	0.656	<0.05	<0.01	<0.01	<0.05	<0.05	0.557	0.692	0.848
CuZnSOD (NU/mg)	<0.001	<0.01	<0.001	<0.05	<0.05	<0.001	<0.001	0.062	<0.001	<0.01	<0.001	<0.001	<0.001	<0.001	0.389	<0.001
MDA (*μ*mol/g)	0.559	<0.001	0.329	<0.01	<0.05	<0.01	<0.01	0.821	<0.001	<0.001	<0.001	<0.01	0.372	<0.05	<0.001	<0.001

GR: glutathione reductase; CAT: catalase; GPX: glutathione peroxidase; SOD: total superoxide dismutase; GST: glutathione-S-transferase; MnSOD: Mn superoxide dismutase; ZnSOD: Zn superoxide dismutase; MDA: malondialdehyde; DJOS: duodenal-jejunal omega switch surgery; HF: high-fat diet; CD: control diet; HF/HF, CD/HF, HF/CD, CD/CD: type of diet 8 weeks before/8 weeks after surgery.

## Data Availability

The data used to support the findings of this study are available from the corresponding author upon request.
