# Corrigendum to “Antioxidant Status in the Soleus Muscle of Sprague-Dawley Rats in Relation to Duodenal-Jejunal Omega Switch and Different Dietary Patterns”

**DOI:** 10.1155/2019/8479861

**Published:** 2019-03-24

**Authors:** Bronisława Skrzep-Poloczek, Dominika Stygar, Elżbieta Chełmecka, Katarzyna Nabrdalik, Ewa Romuk, Jakub Poloczek, Tomasz Sawczyn, Konrad W. Karcz, Janusz Gumprecht

**Affiliations:** ^1^Department of Physiology, School of Medicine with the Division of Dentistry in Zabrze, Medical University of Silesia, Katowice, Poland; ^2^Department of Statistics, Department of Instrumental Analysis, School of Pharmacy with the Division of Laboratory Medicine in Sosnowiec, Medical University of Silesia, Katowice, Poland; ^3^Department of Internal Medicine, Diabetology and Nephrology in Zabrze, Medical University of Silesia, Katowice, Poland; ^4^Department of Biochemistry, School of Medicine with the Division of Dentistry in Zabrze, Medical University of Silesia, Katowice, Poland; ^5^Department of Rehabilitation, 3rd Specialist Hospital in Rybnik, Rybnik, Poland; ^6^Clinic of General, Visceral, Transplantation and Vascular Surgery, Hospital of the Ludwig Maximilian University, Munich, Germany

In the article titled “Antioxidant Status in the Soleus Muscle of Sprague-Dawley Rats in Relation to Duodenal-Jejunal Omega Switch and Different Dietary Patterns” [1], incorrect versions of Figures 2, 3, and 4 were published. The correct versions of the mentioned figures are shown below.

## Figures and Tables

**Figure 1:**
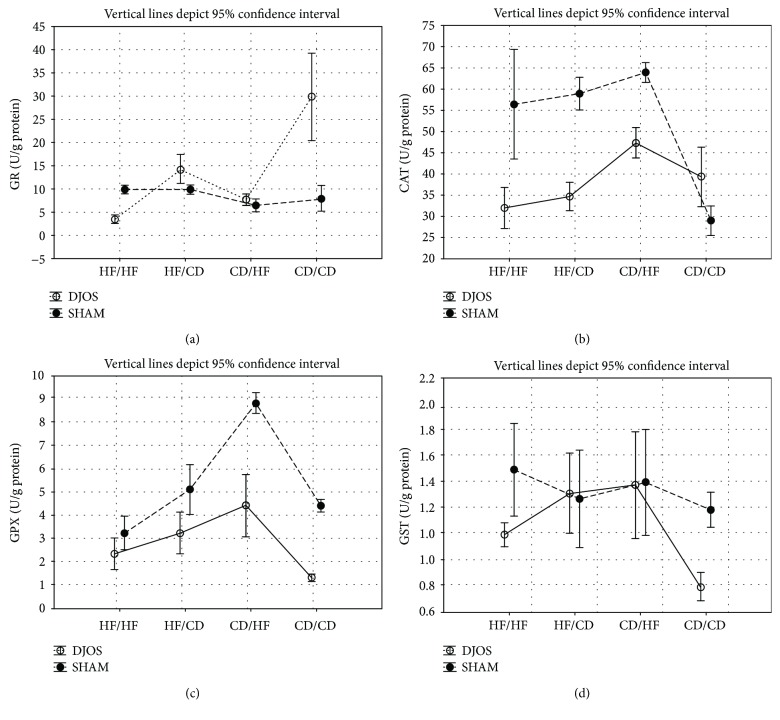
(a) Mean values of GR (IU/g) activity in four groups subjected to different dietary patterns, according to the DJOS and SHAM operation type. Statistical significance was set at *p* < 0.05. Vertical lines depict 95% confidence interval. DJOS: duodenal-jejunal omega switch surgery; HF: high-fat diet; CD: control diet; HF/HF, CD/HF, HF/CD, CD/CD: type of diet 8 weeks before/8 weeks after surgery. (b) Mean values of CAT (IU/g) activity in four groups subjected to different dietary patterns, according to the DJOS and SHAM operation type. Statistical significance was set at *p* < 0.05. Vertical lines depict 95% confidence interval. DJOS: duodenal-jejunal omega switch surgery; HF: high-fat diet; CD: control diet; HF/HF, CD/HF, HF/CD, CD/CD: type of diet 8 weeks before/8 weeks after surgery. (c) Mean values of GPX (IU/g) activity in four groups subjected to different dietary patterns, according to the DJOS and SHAM operation type. Statistical significance was set at *p* < 0.05. Vertical lines depict 95% confidence interval. DJOS: duodenal-jejunal omega switch surgery; HF: high-fat diet; CD: control diet; HF/HF, CD/HF, HF/CD, CD/CD: type of diet 8 weeks before/8 weeks after surgery. (d) Mean values of GST (IU/g) activity in four groups subjected to different dietary patterns, according to the DJOS and SHAM operation type. Statistical significance was set at *p* < 0.05. Vertical lines depict 95% confidence interval. DJOS: duodenal-jejunal omega switch surgery; HF: high-fat diet; CD: control diet; HF/HF, CD/HF, HF/CD, CD/CD: type of diet 8 weeks before/8 weeks after surgery.

**Figure 2:**
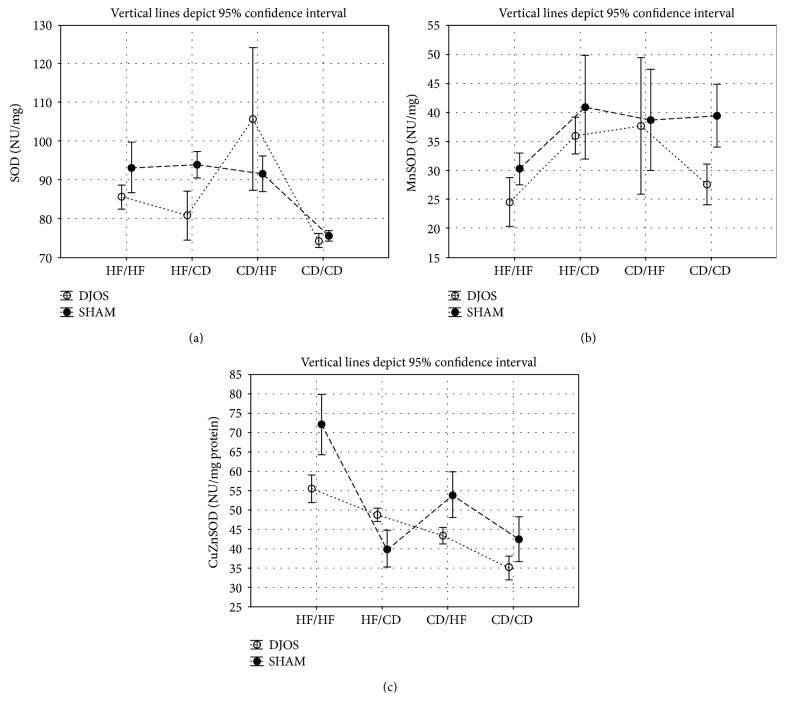
(a) Mean values of SOD (NU/mg) activity in four groups subjected to different dietary patterns, according to the DJOS and SHAM operation type. Statistical significance was set at *p* < 0.05. Vertical lines depict 95% confidence interval. DJOS: duodenal-jejunal omega switch surgery; HF: high-fat diet; CD: control diet; HF/HF, CD/HF, HF/CD, CD/CD: type of diet 8 weeks before/8 weeks after surgery. (b) Mean values of MnSOD (NU/mg) activity in four groups subjected to different dietary patterns, according to the DJOS and SHAM operation type. Statistical significance was set at *p* < 0.05. Vertical lines depict 95% confidence interval. DJOS: duodenal-jejunal omega switch surgery; HF: high-fat diet; CD: control diet; HF/HF, CD/HF, HF/CD, CD/CD: type of diet 8 weeks before/8 weeks after surgery. (c) Mean values of CuZnSOD (NU/mg) activity in four groups subjected to different dietary patterns, according to the DJOS and SHAM operation type. Statistical significance was set at *p* < 0.05. Vertical lines depict 95% confidence interval. DJOS: duodenal-jejunal omega switch surgery; HF: high-fat diet; CD: control diet; HF/HF, CD/HF, HF/CD, CD/CD: type of diet 8 weeks before/8 weeks after surgery.

**Figure 3:**
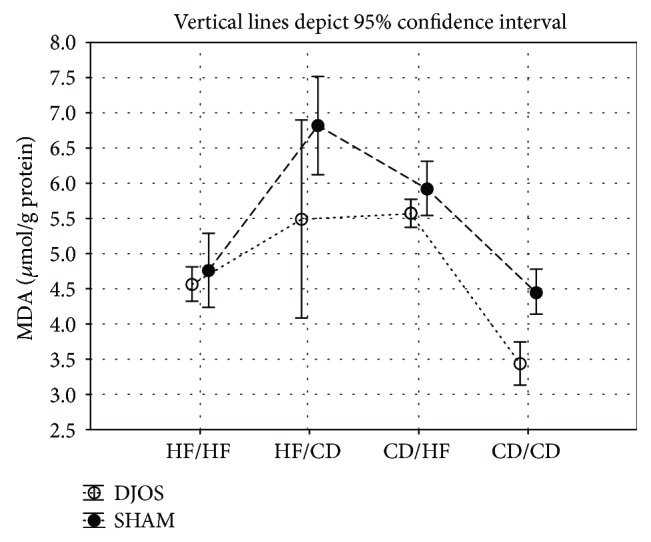
Mean values of MDA (*μ*mol/g) concentration in four groups subjected to different dietary patterns, according to the DJOS and SHAM operation type. Statistical significance was set at *p* < 0.05. Vertical lines depict 95% confidence interval. DJOS: duodenal-jejunal omega switch surgery; HF: high-fat diet; CD: control diet; HF/HF, CD/HF, HF/CD, CD/CD: type of diet 8 weeks before/8 weeks after surgery.

## References

[1] Skrzep-Poloczek B., Stygar D., Chełmecka E. (2018). Antioxidant status in the soleus muscle of Sprague-Dawley rats in relation to duodenal-Jejunal omega switch and different dietary patterns. *Oxidative Medicine and Cellular Longevity*.

